# Trend and Pattern of Herb and Supplement Use in the United States: Results from the 2002, 2007, and 2012 National Health Interview Surveys

**DOI:** 10.1155/2014/872320

**Published:** 2014-12-10

**Authors:** Chung-Hsuen Wu, Chi-Chuan Wang, Meng-Ting Tsai, Wan-Ting Huang, Jae Kennedy

**Affiliations:** ^1^School of Pharmacy, College of Pharmacy, Taipei Medical University, No. 250, Wu-hsing Street, Xinyi District, Taipei 11031, Taiwan; ^2^School of Pharmacy, National Taiwan University, Room 203, 2F, No. 33, Linsen S. Road, Zhongzheng District, Taipei 10050, Taiwan; ^3^Department of Pharmacy, Taipei Medical University Hospital, Taipei Medical University, No. 250, Wu-hsing Street, Xinyi District, Taipei 11031, Taiwan; ^4^Department of Pharmacy, Shuang Ho Hospital, Taipei Medical University, No. 250, Wu-hsing Street, Xinyi District, Taipei 11031, Taiwan; ^5^Department of Health Policy and Administration, Washington State University, SCLS 141, 668 N. Riverpoint Boulevard, Spokane, WA 99210, USA

## Abstract

*Background*. In 1990s, complementary and alternative medicine (CAM), including use of herbs and supplements, gained popularity in the United States. However, more recent surveys suggest that demand for herbs and supplements has stabilized. *Objective*. This study examined the prevalence, patterns, and changes in herb and supplement use among the US adults, using the 2002, 2007, and 2012 National Health Interview Surveys (NHIS). *Methods*. Weighted population estimates were derived from three complementary and alternative medicine supplements to the NHIS. Prevalence rates for herb and supplement use were compared, using Wald chi-square tests to measure changes between years. *Results*. An estimated 40.6 million US adults reported herb and supplement use in 2012. However, the rate of herb and supplement use dropped from 18.9% in 2002 to 17.9% in 2007 and 2012 (*P* < 0.05). This decline in use was more pronounced among women, racial or ethnic minorities, and adults with low incomes. *Conclusion*. Herb and supplements use remains common in the USA, but adult use rates are on the decline. It is still important for health care providers to ask patients about herb and supplement use.

## 1. Introduction

In 1990s, complementary and alternative medicine (CAM) gained popularity in the United States [1–4]. Herbal preparations and dietary supplements were one of the most widely used forms of CAM [1, 5–7], and their popularity increased significantly during this period. For example, Eisenberg et al. estimated the prevalence of adults using herbs increased from 2.5% in 1990 to 12.1% in 1997 [1]. More recently, about 18% of the US population (over 38 million US adults) reported using herbal preparations and dietary supplements in 2007 [8, 9]. From an economic perspective, there was an approximate 20% herbal sale growth rate per year in retail pharmacy [4]. Regarding the popularity, several population groups also reported relatively high rates of herb and supplement use, including women [8, 10–17], middle aged adults [10, 16], Caucasians [10, 11, 14, 15], Asians [12, 18], and Latinos [19].

The National Center for Health Statistics began to conduct a periodic national survey of CAM use in 1997, adding a supplement to the National Health Interview Survey (NHIS) every five years [20–22]. In a previous study of the 2002 and 2007 NHIS, we found that the number of herb and supplement users increased from 38.1 million in 2002 to 38.8 million in 2007, but 12-month use rates decreased from 18.9% in 2002 to 17.9% in 2007 [9]. It is essential to update the use pattern and to better understand the trend change of herb and supplement use.

A new CAM survey was released in the 2012 NHIS, and the purpose of this study was to examine the prevalence, pattern, and trend of the herb and supplement use among the US adults over a 10-year period.

## 2. Methods

### 2.1. Data Source

The National Health Interview Survey (NHIS) is a publicly available survey conducted by the National Center for Health Statistics (NCHS) [22]. The NHIS collects information on a broad range of health topics, as well as demographic and socioeconomic characteristics for the US noninstitutionalized population [22]. The cross-sectional interview survey, which is a statistically representative sample of the US population, includes four main components: the household, family, sample child, and sample adult core surveys. In 2002, 2007, and 2012, the NCHS added supplementary questionnaires, referred to as Adult Complementary and Alternative Medicine File (ALT), to the sample adult core surveys to collect information on various types of complementary and alternative medicine use among respondents [22]. The response rates were 74.3% in 2002, 67.8% in 2007, and 61.2% in 2012 [20–22].

### 2.2. Dependent Variables, Herb and Supplement Use

In 2002, the survey question phrased was “during the past 12 months, did you use natural herbs for your own health or treatment?” In 2007, the question was phrased as “during the past 12 months, have you ever taken any herbal supplements listed on this card for yourself?” In 2012, the question was similar to the question in 2007 but with fewer kinds of herbal preparations and dietary supplements on the list. The question was phrased as “during the past 12 months, have you taken any herbal or other nonvitamin supplements listed on this card for yourself?” In the 2007 and 2012 surveys, respondents were also asked if they have taken any herbal preparations and dietary supplements during the past 30 days and then asked to identify specific herbal preparations and dietary supplements from the list. The 2007 and 2012 surveys were independent. Some of herbal preparations and dietary supplements were listed in both years, but the composition of this list changed from year to year.

### 2.3. Covariates

Selected characteristics that may be associated with herb and supplement use were compared between years (2002 versus 2012 and 2007 versus 2012) to identify changes in patterns and trends. Characteristics included age (<65 versus ≥65), gender, race (white versus nonwhite), ethnicity (Hispanic versus non-Hispanic), years of education (<12 years, 12 years, and >12 years), family income (less than $35,000 versus $35,000 or more), self-assessed health status (excellent to good versus fair to poor), whether respondents had health insurance coverage (yes versus no), and whether respondents reported they could not afford prescriptions (yes versus no).

### 2.4. Statistical Analysis

Two major statistical analysis steps were in our study. First, we used descriptive statistics to estimate the prevalence of herb and supplements use, frequency of respondent characteristics, and the frequency of a specific herb and supplement use among herb and supplement users in the preceding year or past month. Second, we evaluated trend changes of herb and supplement use as well as the user frequency of respondent characteristics between years. The trend changes of herb and supplement use were compared between 2002 and 2012 as well as 2007 and 2012. The trend difference of the respondent characteristics was also compared between years.

There are some variations in the questionnaires used to assess herb and supplement use in 2002, 2007, and 2012. The recall period for specific herb and supplement use was 12 months in 2002 and 30 days in 2007, while in the 2012 questionnaire, the specific types of herbal preparations and dietary supplements used were asked for both the past 12 months and 30 days. Due to the variation of the questionnaire design, we then only listed herb and supplement use among current users who reported use of herbal preparations and dietary supplements in the past 30 days in 2007 and 2012 surveys.

Data management and statistical analyses were performed using SAS v. 9.3 version [23]. The survey sample design of the NHIS is with multistage, stratification, and clusters [22]. All analyses and estimates were adjusted for complex sample design using PROC SURVEY procedure in SAS v. 9.3 to represent the US population. Taylor series linearization methods were used for the variance estimation, and the Wald chi-square tests were used to evaluate the changes between years.

## 3. Results

Table 1 shows the prevalence of herb and supplement use among the US adults in 2002, 2007, and 2012. While the estimated number of adults who ever utilized herbal preparations and dietary supplements increased from 50.6 million in 2002 to 53.6 million in 2012, the proportion of herb and supplement users actually went down slightly from 25.1% to 23.6% (−1.5%, *P* < 0.01). The one-year prevalence of the herb and supplement use significantly decreased from 2002 to 2012 (18.9% to 17.9%, *P* < 0.05), but no significant change was found between 2007 and 2012.

Table 2 compares the population attributes across years among adults who used the herbal preparations and dietary supplements in the past 12 months. Significant drops in the prevalence of herb and supplement use between 2002 and 2012 were found among adults aged 18 to 64 years (20.0% to 17.8%, *P* < 0.001), females (21.0% to 19.2%, *P* < 0.001), nonwhites (17.7% to 12.9%, *P* < 0.001), Hispanics (17.4% to 11.3%, *P* < 0.001), adults with 12 years of education (15.3% to 13.2%, *P* < 0.01), adults with annual family income less than $35,000 (17.2% to 13.4%, *P* < 0.001), adults with family income ≥$35,000 (21.9% to 20.5%, *P* < 0.05), adults with fair to poor health (16.7% to 14.5%, *P* < 0.05), adults without health insurance (18.2% to 13.8%, *P* < 0.001), and adults who could not afford prescriptions (26.9% to 20.9%, *P* < 0.001). The only subgroup with an increased rate of herb and supplement use was adults aged 65 years or older, from 13.2% in 2002 to 18.2% in 2012 (*P* < 0.001). The rates of herb and supplement use did not change significantly among the selected subgroups between 2007 and 2012, with only one exception: adults with fair to poor health status (17.2% to 14.5%, *P* < 0.05).

Table 3 summarizes the types and frequencies of herb and supplement use among current users in 2007 and 2012. Chondroitin, coenzyme Q-10, Echinacea, fish oil, garlic supplements, ginkgo, ginseng, and glucosamine were the most frequently used herbal preparations and dietary supplements across the years although there is a decrease in the use of chondroitin, Echinacea, garlic supplements, ginkgo, and ginseng. In contrast, coenzyme Q-10, fish oil, melatonin, and probiotics seemed to gain popularity in 2012.

## 4. Discussion

We found that the one-year prevalence of herb and supplement use declined over the previous decade, even though the absolute number of adult herb and supplement users increased from 38.2 million in 2002 to 40.6 million in 2012. This decline in use was more pronounced among women, racial or ethnic minorities, and adults with low incomes. This change may reflect changing consumer tastes and, more recently, improved access to conventional therapies due to federal and state health reforms.

This observed decline may be due in part to the narrower operational definitions of herb and supplement use in the later (2007 and 2012) surveys. While the 2002 survey asked a general question about herb and supplement use, later surveys made reference to a specific list of herbal preparations or dietary supplements. Moreover, the 2007 survey used a flash card with a list of 45 different herbal preparations or dietary supplements, while only 22 were listed on a flash card in the 2012 survey.

We also found that a growing number of adults over the age of 65 reported herb and supplement use. Like our previous study [9], we remained to observe that chondroitin, glucosamine, fish oil, coenzyme Q-10, garlic supplements, ginkgo, ginseng, and Echinacea were popular herbal preparations or dietary supplements in both 2007 and 2012 surveys. The popularity may be due to a growing aged population with a strong demand of treatment for cardiovascular diseases (fish oil, coenzyme Q-10, garlic supplements, ginkgo, and ginseng) and joint problems (glucosamine and chondroitin). Although the efficacy of herbal preparations and dietary supplements remains controversial, users who are in favor of personal health control often have strong beliefs that herbal preparations and dietary supplements are natural and with fewer side effects [24, 25].

The herb and supplement section in the 2007 NHIS ALT survey is a relatively comprehensive survey section that can identify and evaluate the change of herb and supplement use. However, fewer questionnaires were included in the herb and supplement section in the 2012 NHIS ALT survey. Regarding the popularity of herb and supplement use found in our study, reasons for taking herbal preparations and dietary supplements are necessary to be included in the next NHIS ALT survey in order to thoroughly understand reasons behind taking herbal preparations and dietary supplements. Furthermore, questionnaires regarding herb-drug interactions or health beliefs of herb and supplement use are also necessary in order to provide safety information for health care providers. Evidence derived from these additional questionnaires can provide rich information for health care providers to better communicate the use of herbal preparations and dietary supplements with their patients.

There were several limitations that needed to be mentioned in this study. The NHIS was a large health survey with secondary data but was still inherent to recall bias. The dependent variable was defined slightly different each year, which makes the interpretation and comparison of the study results become difficult. The use of flashcards with different numbers of herbal preparations and dietary supplements listed in different years could underestimate the prevalence. For example, St. John's Wort, a popular herbal medicine used to treat depression, was listed in the 2002 survey but it was not listed in the 2012 survey. Despite these limitations, the NHIS is still the most comprehensive US national survey for CAM use. Our study provided the most updated data and examined the trend of herb and supplements use in the past 10 years.

## 5. Conclusion

Herb and supplement use remains common in the USA, but the 12-month use rate is on the decline. Discussing CAM use with patients continues to be the priority for health care providers. Future research should investigate the decline trend of herb and supplements use among racial/ethnic minorities and assess the impact of health policy changes on herb and supplement use among people with lower socioeconomic status.

## Figures and Tables

**Table 1:** Prevalence of herb and supplement use among the US adult population in 2002, 2007, and 2012.

Sample size and population estimates	2002			2007			2012			Percentage change	Percentage change
	Sample *n*	Est. *N*	%^a^	Sample *n*	Est. *N*	%^a^	Sample *n*	Est. *N*	%^a^	(2002 and 2012)	(2007 and 2012)
Total	**30,427**	**201,674,075**	**100.0%**	**22,657**	**216,781,365**	**100.0%**	**34,525**	**234,920,670**	**100.0%**		
Ever taken any herbal preparations and dietary supplements	7,655	50,613,051	25.1%	5,600	55,090,245	25.4%	7,864	53,571,888	23.6%	−1.5%^**^	−1.8%^**^
Took herbal preparations and dietary supplements in past 12 months	5,787	38,182,843	18.9%	3,982	38,797,215	17.9%	5,974	40,579,309	17.9%	−1.0%^*^	0.0%
Took herbal preparations and dietary supplements in past 30 days^b^	na	na	na	2,932	28,059,181	12.9%	4,491	30,113,788	13.3%	na	0.3%

Data source: National Center for Health Statistics, 2002, 2007, and 2012 NHIS Adult Complementary and Alternative Medicine File [20–22].

Note: proportions based on weighted prevalence estimates, *P* values for Wald chi-square tests.

^a^Column percentage.

^b^The question of the current herb use was only asked in 2007 and 2012 NHIS.

na: not available.

^*^ *P* < 0.05.

^**^ *P* < 0.01.

**Table 2:** Changes of patient characteristics of herb and supplement users.

Patient characteristics associated with herb and supplement use	2002		2007		2012		Percentage change	Percentage change
	Est. *N*	%^a^	Est. *N*	%^a^	Est. *N*	%^a^	(2002 and 2012)	(2007 and 2012)
Took herbal preparations and dietary supplements, past 12 months	38,182,843	18.9%	38,797,215	17.9%	40,579,309	17.9%	−1.0%	0.0%

Age								
18–64	33,929,605	20.0%	31,982,187	17.6%	33,260,541	17.8%	−2.2%^***^	0.2%
65+	4,253,238	13.2%	6,815,028	19.5%	7,318,768	18.2%	5.0%^***^	−1.4%
Gender								
Male	16,153,313	16.7%	16,905,038	16.2%	17,972,393	16.4%	−0.3%	0.3%
Female	22,029,530	21.0%	21,892,177	19.5%	22,606,916	19.2%	−1.8%^**^	−0.3%
Race								
White	31,584,163	19.2%	33,923,386	19.1%	35,054,977	19.0%	−0.2%	−0.1%
Nonwhite	6,598,680	17.7%	4,873,829	12.4%	5,524,332	12.9%	−4.8%^***^	0.5%
Ethnicity								
Non-Hispanic	34,319,989	19.1%	35,970,017	19.2%	36,717,730	19.0%	−0.1%	−0.1%
Hispanic	3,862,854	17.4%	2,827,198	9.7%	3,861,579	11.3%	−6.0%^***^	1.6%
Education								
<12 years	3,501,656	10.6%	3,681,423	9.3%	3,523,914	9.2%	−1.4%	−0.2%
12 years	8,084,698	15.3%	8,031,004	14.3%	6,946,425	13.2%	−2.0%^**^	−1.1%
>12 years	26,596,489	23.0%	27,084,788	22.3%	30,022,074	22.2%	−0.8%	−0.1%
Annual family income								
Less than $35,000	9,918,920	17.2%	9,616,154	14.2%	9,631,584	13.4%	−3.7%^***^	−0.8%
$35,000 or more	21,055,257	21.9%	26,073,787	20.5%	29,156,013	20.5%	−1.4%^*^	0.0%
Self-assessed health status								
Excellent-good	34,065,198	19.3%	33,854,214	18.0%	36,342,227	18.4%	−0.9%	0.4%
Fair-poor	4,073,931	16.7%	4,931,346	17.2%	4,229,285	14.5%	−2.1%^*^	−2.7%^*^
Health insurance coverage								
Yes	32,422,709	19.1%	33,401,786	18.6%	35,221,068	18.7%	−0.4%	0.1%
No	5,633,751	18.2%	5,279,896	16.4%	5,263,261	13.8%	−4.4%^***^	−2.6%
Could not fill prescription drug in past 12 months due to cost								
Yes	3,912,807	26.9%	4,359,261	23.1%	3,931,622	20.9%	−6.0%^***^	−2.2%
No	34,231,673	18.3%	34,437,954	17.4%	36,643,776	17.6%	−0.7%	0.2%

Data source: National Center for Health Statistics, 2002 and 2007 NHIS Adult Complementary and Alternative Medicine File [20, 21].

Note: proportions based on weighted prevalence estimates, *P* values for Wald chi-square tests.

^a^Row percentage.

^*^ *P* < 0.05.

^**^ *P* < 0.01.

^***^ *P* < 0.001.

**Table 3:** Specific types of herb and supplement use among the US adult population in 2007 and 2012.

Herb and supplement use	2007		2012	
	Est. *N*	%^a^	Est. *N*	%^a^
Taken herbal preparations and dietary supplements, past 30 days (2007, 2012)	28,059,181	100.0%	30,113,788	100.0%

Acai pills or gelcaps	na	na	1,169,912	3.9%
Androstenedione	57,402	0.2%	na	na
Bee pollen or royal jelly	na	na	1,054,869	3.5%
Black cohosh	827,675	2.9%	na	na
Bladder wrack/kelp	na	na	1,169,912	3.9%
Carnitine	404,001	1.4%	na	na
Chasteberry	127,391	0.5%	na	na
Chondroitin	3,389,911	12.1%	2,815,929	9.4%
Coenzyme Q-10	2,691,440	9.6%	3,264,650	10.8%
Comfrey	164,047	0.6%	na	na
Conjugated linolenic acid (CLA)	334,340	1.2%	na	na
Cranberry (pills, gelcaps)	1,560,383	5.6%	1,934,165	6.4%
Creatine	843,385	3.0%	na	na
DHEA	645,179	2.3%	na	na
Digestive enzymes (lactaid)	na	na	1,639,041	5.4%
Dong quai/don gui tong kuei	na	na	na	na
Echinacea	4,848,163	17.3%	2,261,040	7.5%
Ephedra (Ma huang)	314,161	1.1%	na	na
Evening primrose	555,303	2.0%	na	na
Feverfew	146,723	0.5%	na	na
Fiber or psyllium (pills or powder)	1,791,164	6.4%	na	na
Fish oil/omega 3/DHA	10,922,801	38.9%	18,848,204	62.6%
Flaxseed oil or pills	4,416,216	15.7%	na	na
Garlic supplements (pills, gelcaps)	3,278,373	11.7%	1,926,964	6.4%
Ginger pills/gelcaps	847,658	3.0%	na	na
*Ginkgobiloba *	2,977,104	10.6%	1,619,090	5.4%
Ginseng	3,345,357	11.9%	1,751,717	5.8%
Glucosamine	6,132,094	21.9%	5,521,173	18.3%
Goldenseal	825,015	2.9%	na	na
Guarana	498,254	1.8%	na	na
Grape seed extract	1,214,475	4.3%	na	na
Green tea pills (not brewed tea)	1,527,759	5.4%	na	na
EGCG (pills)	97,369	0.3%	na	na
Green tea pills or EGCG pill	na	na	1,502,627	5.0%
Hawthorn	307,645	1.1%	na	na
Horny goat weed	136,747	0.5%	na	na
Kava kava	356,802	1.3%	na	na
Lecithin	903,188	3.2%	na	na
Lutein	1,046,629	3.7%	na	na
Lycopene	821,468	2.9%	na	na
Melatonin	1,295,762	4.6%	3,065,428	10.2%
MSM (methylsulfonylmethane)	1,311,824	4.7%	1,050,502	3.5%
Milk thistle	1,000,750	3.6%	987,957	3.3%
Prebiotics or probiotics	865,092	3.1%	3,857,228	12.8%
SAM-e	212,233	0.8%	414,563	1.4%
Saw palmetto	1,681,668	6.0%	988,070	3.3%
Senna	185,671	0.7%	na	na
Soy supplements/isoflavones	1,362,529	4.9%	na	na
St. John's Wort	933,060	3.3%	na	na
Valerian	877,107	3.1%	800,946	2.7%

Data source: National Center for Health Statistics 2007 and 2012 NHIS Adult Complementary and Alternative Medicine File [21, 22].

^a^Weighted estimates of column percentage in taken herb and supplement in past 30 days.

na: the names of the herbal preparations and dietary supplements were not listed in the survey questionnaire.

## References

[1] Eisenberg D. M., Davis R. B., Ettner S. L., Appel S., Wilkey S., Van Rompay M., Kessler R. C. (1998). Trends in alternative medicine use in the United States, 1990–1997: results of a follow-up national survey. *The Journal of the American Medical Association*.

[2] Eisenberg D. M., Kessler R. C., Foster C., Norlock F. E., Calkins D. R., Delbanco T. L. (1993). Unconventional medicine in the United States: prevalence, costs, and patterns of use. *The New England Journal of Medicine*.

[3] Eisenberg D. M., Kessler R. C., Van Rompay M. I., Kaptchuk T. J., Wilkey S. A., Appel S., Davis R. B. (2001). Perceptions about complementary therapies relative to conventional therapies among adults who use both: results from a national survey. *Annals of Internal Medicine*.

[4] Kessler R. C., Davis R. B., Foster D. F., Van Rompay M. I., Walters E. E., Wilkey S. A., Kaptchuk T. J., Eisenberg D. M. (2001). Long-term trends in the use of complementary and alternative medical therapies in the United States. *Annals of Internal Medicine*.

[5] Tindle H. A., Davis R. B., Phillips R. S., Eisenberg D. M. (2005). Trends in use of complementary and alternative medicine by us adults: 1997–2002. *Alternative Therapies in Health and Medicine*.

[6] Yu S. M., Ghandour R. M., Huang Z. J. (2004). Herbal supplement use among US women, 2000. *Journal of the American Medical Women's Association*.

[7] Alkhateeb F. M., Doucette W. R., Ganther-Urmie J. M. (2006). Influences on consumer spending for herbal products. *Research in Social and Administrative Pharmacy*.

[8] Kennedy J. (2005). Herb and supplement use in the US adult population. *Clinical Therapeutics*.

[9] Wu C.-H., Wang C.-C., Kennedy J. (2011). Changes in herb and dietary supplement use in the US adult population: a comparison of the 2002 and 2007 national health interview surveys. *Clinical Therapeutics*.

[10] Schaffer D. M., Gordon N. P., Jensen C. D., Avins A. L. (2003). Nonvitamin, nonmineral supplement use over a 12-month period by adult members of a large health maintenance organization. *Journal of the American Dietetic Association*.

[11] Kelly J. P., Kaufman D. W., Kelley K., Rosenberg L., Mitchell A. A. (2006). Use of herbal/natural supplements according to racial/ethnic group. *Journal of Alternative and Complementary Medicine*.

[12] Tanaka M. J., Gryzlak B. M., Zimmerman M. B., Nisly N. L., Wallace R. B. (2008). Patterns of natural herb use by Asian and Pacific Islanders. *Ethnicity and Health*.

[13] Dailey R. K., Neale A. V., Northrup J., West P., Schwartz K. L. (2003). Herbal product use and menopause symptom relief in primary care patients: a metronet study. *Journal of Women's Health*.

[14] Mackenzie E. R., Taylor L., Bloom B. S., Hufford D. J., Johnson J. C. (2003). Ethnic minority use of complementary and alternative medicine (CAM): a national probability survey of CAM utilizers. *Alternative Therapies in Health and Medicine*.

[15] Hung O. L., Shih R. D., Chiang W. K., Nelson L. S., Hoffman R. S., Goldfrank L. R. (1997). Herbal preparation use among urban emergency department patients. *Academic Emergency Medicine*.

[16] Klepser T. B., Doucette W. R., Horton M. R. (2000). Assessment of patients' perceptions and beliefs regarding herbal therapies. *Pharmacotherapy*.

[17] Harnack L. J., Rydell S. A., Stang J. (2001). Prevalence of use of herbal products by adults in the Minneapolis/St Paul, Minn, metropolitan area. *Mayo Clinic Proceedings*.

[18] Bair Y. A., Gold E. B., Greendale G. A. (2002). Ethnic differences in use of complementary and alternative medicine at midlife: longitudinal results from SWAN participants. *American Journal of Public Health*.

[19] Howell L., Kochhar K., Saywell R. M., Zollinger T., Koehler J., Mandzuk C., Sutton B., Sevilla-Martir J., Allen D. (2006). Use of herbal remedies by Hispanic patients: do they inform their physician?. *Journal of the American Board of Family Medicine*.

[20] National Center for Health Statistics (NCHS) (2003). *2002 National Health Interview Survey (NHIS) Public Use Data Release: NHIS Survey Description*.

[21] National Center for Health Statistics (NCHS) (2008). *2007 National Health Interview Survey (NHIS) Public Use Data Release: NHIS Survey Descripition*.

[22] National Center for Health Statistics (NCHS) (2013). *2012 National Health Interview Survey (NHIS) Public Use Data Release: NHIS Survey Descripition*.

[23] SAS Institute (2011). *SAS/STAT 9.3 User's Guide*.

[24] Cheung C. K., Wyman J. F., Halcon L. L. (2007). Use of complementary and alternative therapies in community-dwelling older adults. *The Journal of Alternative and Complementary Medicine*.

[25] Astin J. A. (1998). Why patients use alternative medicine: results of a national study. *Journal of the American Medical Association*.

